# Insulin activates parasympathetic hepatic-related neurons of the paraventricular nucleus of the hypothalamus through mTOR signaling

**DOI:** 10.1152/jn.00284.2024

**Published:** 2024-12-12

**Authors:** Karoline Martins dos Santos, Sandy E. Saunders, Vagner R. Antunes, Carie R. Boychuk

**Affiliations:** ^1^Department of Cellular and Integrative Physiology, Long School of Medicine, University of Texas Health San Antonio, San Antonio, Texas, United States; ^2^Department of Physiology & Biophysics, Institute of Biomedical Sciences, University of São Paulo, São Paulo, Brazil; ^3^Dalton Cardiovascular Research Center, Department of Biomedical Sciences, College of Veterinary Medicine, University of Missouri, Columbia, Missouri, United States

**Keywords:** hypothalamus, insulin, liver, parasympathetic, paraventricular

## Abstract

Integration of autonomic and metabolic regulation, including hepatic function, is a critical role played by the brain’s hypothalamic region. Specifically, the paraventricular nucleus of the hypothalamus (PVN) regulates autonomic functions related to metabolism, such as hepatic glucose production. Although insulin can act directly on hepatic tissue to inhibit hepatic glucose production, recent evidence implicates that central actions of insulin within PVN also regulate glucose metabolism. However, specific central circuits responsible for insulin signaling with relation to hepatic regulation are poorly understood. As a heterogeneous nucleus essential to controlling parasympathetic motor output with notable expression of insulin receptors, PVN is an appealing target for insulin-dependent modulation of parasympathetic activity. Here, we tested the hypothesis that insulin activates hepatic-related PVN (PVN^hepatic^) neurons through a parasympathetic pathway. Using transsynaptic retrograde tracing, labeling within PVN was first identified 24 h after its expression in the dorsal motor nucleus of the vagus (DMV) and 72 h after hepatic injection. Critically, nearly all labeling in medial PVN was abolished after a left vagotomy, indicating that PVN^hepatic^ neurons in this region are part of a central circuit innervating parasympathetic motor neurons. Insulin also significantly increased the firing frequency of PVN^hepatic^ neurons in this subregion. Mechanistically, rapamycin pretreatment inhibited insulin-dependent activation of PVN^hepatic^ neurons. Therefore, central insulin signaling can activate a subset of PVN^hepatic^ neurons that are part of a unique parasympathetic network in control of hepatic function. Taken together, PVN^hepatic^ neurons related to parasympathetic output regulation could serve as a key central network in insulin’s ability to control hepatic functions.

**NEW & NOTEWORTHY** Increased peripheral insulin concentrations are known to decrease hepatic glucose production through both direct actions on hepatocytes and central autonomic networks. Despite this understanding, how (and in which brain regions) insulin exerts its action is still obscure. Here, we demonstrate that insulin activates parasympathetic hepatic-related PVN neurons (PVN^hepatic^) and that this effect relies on mammalian target of rapamycin (mTOR) signaling, suggesting that insulin modulates hepatic function through autonomic pathways involving insulin receptor intracellular signaling cascades.

Listen to this article’s corresponding podcast at https://jneurophysiol.podbean.com/e/jnp-podcasts-unlocking-insulins-mechanism-activation-of-parasympathetic-hepatic-neurons-via-mtor-signaling/ .

## Graphical abstract

**Figure:**
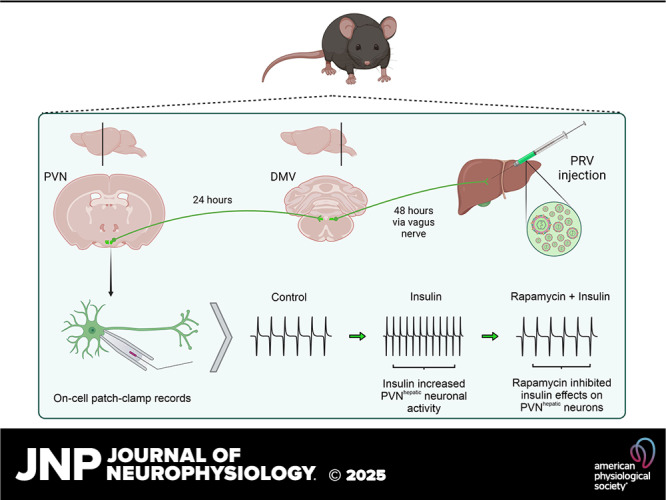

## INTRODUCTION

One major life-threatening condition driving mammalian evolution is glucose availability, and as such glucose homeostasis must be tightly regulated (1). It is well appreciated that blood glucose level is maintained through the regulation of hepatic tissues, specifically modulation of glycogen production and hepatic vein glucose concentration (2). Although the brain’s role in glucose homeostasis was first described by Claude Bernard (3), a more recent body of evidence now firmly establishes autonomic nervous system (ANS) activity as a central mechanism to further modulate, or fine-tune, glucose homeostasis (4, 5) as it relates to hepatic function.

The ANS is regulated via preautonomic hypothalamic neurons projecting to various brainstem nuclei. The liver receives innervation from both sympathetic and parasympathetic nerves. Sympathetic splanchnic innervation to the liver originates from neurons located in the celiac and superior mesenteric ganglia. On the other hand, parasympathetic innervation to the liver originates from preganglionic motor neurons situated in the brainstem’s dorsal motor nucleus of the vagus (DMV) (6), with axonal branching exclusively from the left vagus nerve (7).

The pancreatic peptide, insulin, despite its classical actions on peripheral organs to tightly control glycemia, also plays a significant physiological role in the control of hepatic glucose homeostasis via its modulation of central autonomic circuits (8). For instance, central insulin administration leads to reductions in glucose production and decreases in food intake (9, 10). However, the specific brain sites and the neuronal network involved in the central action of insulin to mediate central parasympathetic control of hepatic glucose homeostasis are still up for debate, and likely include circuits upstream of liver-projecting DMV neurons.

The hypothalamus plays a critical role in the integration of autonomic and metabolic regulation (11), including hepatic function (6, 12). Of particular importance, the paraventricular nucleus of the hypothalamus (PVN) is a heterogeneous nucleus with monosynaptic projections to DMV that are critical in the control of parasympathetic motor output (13, 14). PVN neurons also express insulin receptors (15) in neurons retrogradely labeled from hepatic tissue (16). Taken together, hepatic-related PVN neurons (PVN^hepatic^) are an attractive target for insulin-dependent modulation of parasympathetic activity. However, the mechanisms underlying insulin’s influence on PVN^hepatic^ neuronal activity lack clarity and whether PVN contains neurons related to regulation of parasympathetic motor output remains unknown.

Since PVN neurons send direct glutamatergic connections to DMV (17, 18) where vagal preganglionic motor neurons are located with efferent projection to hepatic tissue, we hypothesized that insulin acts to increase parasympathetic-related PVN^hepatic^ neuron activity. To test this hypothesis, we used pseudo-rabies virus (PRV) transsynaptic retrograde tracing paired with immunohistochemical techniques to identify potential parasympathetic-related PVN^hepatic^ neurons, and their peptide phenotype(s). We also used electrophysiological patch-clamp techniques to investigate the action(s) of insulin on these neurons.

## EXPERIMENTAL PROCEDURES

### Animals

All procedures were in accordance with National Institute for Health (NIH) standards for the care and use of laboratory animals and protocols were approved by the University of Texas Health San Antonio (UTHSA) animal welfare committee (Animal Care and Use Committees; IACUC; AALC No. D16-00224). All experiments were performed on young male and female adult C57/Bl6J mice (8 wk, 18–25 g). An inclusive design for sex was used as males and females were included to ensure equal number of both sexes in all groups. Breeding mice were first purchased from Jackson Research Laboratory and were an established in-house colony at the University of Texas Health San Antonio (UTHSA) vivarium. Where indicated, a subset of experiments used male and female mice expressing Cre-recombinase under the corticotrophin-releasing hormone promotor (CRH-Cre mice, 8 wk, 18–25 g). Animals were housed within a central facility of UTHSA in a 14:10 light-dark cycle and received chow and water ad libitum. All attempts were made to minimize animal discomfort and pain.

### Retrograde Labeling of PVN^hepatic^ Neurons

A pseudo-rabies virus (PRV-152) strain isogenic with the attenuated PRV Bartha, constructed to report enhanced green fluorescent protein (eGFP; 7.38*e*9 pfu/mL; Center of Neuroanatomy with Neutropic Viruses, University of Princeton) was used to retrogradely label neurons related to hepatic regulating circuits. Identification of PRV-152+ neurons using the expression of eGFP would implicate neurons as transsynaptically coupled to hepatic tissue when PRV-152 is injected into the liver. Animals were anesthetized with isoflurane (5% induction, then maintained at 2% O_2_-inspired air) until lack of withdrawal reflex (i.e., lack of a toe pinch response) and maintained at 37°C with a heating pad. Surgical sites were shaved and aseptically prepared with betadine and alcohol. A laparotomy was performed to expose the liver. Using a Hamilton syringe (100 µL) with a PE10 extension and a 26-gauge needle, 40 µL of PRV-152 (20 sites, 2 µL each) was injected into the hepatic parenchyma (right, middle, and left lobes). Each injection was done over the course of 1 min and the needle remained in place for 1 min after injection. All injection sites were immediately sealed with a drop of liquid bandage (Nexcare No Sting Liquid Bandage, 3M Consumer Health Care, St. Paul, MN), and the abdominal cavity was rinsed thoroughly with sterile saline to prevent the virus from leaking into the abdominal cavity and labeling other organs. In a subset of control animals (*n* = 3), PRV was nonselectively injected into the abdominal cavity and rinsed as stated earlier. After 72 h, no PRV+ labeling was seen in DMV of this subset of mice. The abdominal wall was then sutured with 5-0 suture and the skin was closed with staples. Animals were monitored until ambulatory and housed in a biosafety level 2 vivarium (BSL2) for up to 96 h postinoculation. All animals received subcutaneous injections of buprenorphine (0.1 mg/kg) and carprofen (10 mg/kg) for two days for pain management. To determine the impact of insulin specifically on PVN^hepatic^ neurons in the parasympathetic pathway, we first developed a timeline for retrograde labeling of PVN^hepatic^ neurons based on endogenous expression of the eGFP reporter. Based on previous reports (19, 20), animals were euthanized at 48 h, 72 h, and 96 h post PRV-152 after overdose by isoflurane inhalation. Mice were then transcardially perfused with 20 mL of cold phosphate buffer solution (PBS; with 0.1 mL of heparin) and 20 mL of cold 4% paraformaldehyde (PFA) solution. Brains were removed, kept overnight in a 4% PFA solution, and cryoprotected in 30% sucrose for two days (until tissue sunk). Brains were then sectioned at 40 µm on a cryostat (CM1860, Leica Biosystems, Buffalo Groove, IL). Every sixth section was mounted on charged slides (SuperFrost Plus Microscope Slides, Fisher Scientific, Pittsburgh, PA) and coverslipped with Vectabond (Vector Laboratories, Newark, CA). As a result, one-sixth of each brain was imaged, using either an Olympus BX43 equipped with a Retiga R6 (Teledyne Imaging, Tucson, AZ) or an All-in-One Fluorescence (Keyence BZ-X800E, Itasca, IL) microscope. Cells expressing endogenous fluorescence were manually counted on Fiji Software (ImageJ) using a cell-counter plug-in and compared for each time-point after PRV injection. PVN subregions were examined based on an atlas, *The Mouse Brain in Stereotaxic Coordinates* (21), and compared by rostrocaudal distribution.

### Vagotomy

Since PVN neurons also project directly to the intermediolateral column or rostral ventral lateral medulla to control sympathetic motor output (i.e., not involving DMV), a vagotomy was performed before PRV-152 hepatic injection in a subset of animals. Mice were anesthetized with isoflurane (5% induction, then maintained at 2% O_2_-inspired air) until lack of withdrawal reflex (i.e., lack of a toe pinch response) and maintained at 37°C with a heating pad. The ventral surface of the neck was aseptically prepared with betadine and alcohol. An incision was made just lateral to the midline on the left side and tissue was bluntly dissected to expose the carotid sheath. The left branch of the vagus nerve was then carefully dissected from the carotid artery and a 3-mm section was removed. The incision was then closed with 5-0 suture, and PRV-152 hepatic injection was completed as described earlier.

### Immunohistochemical Phenotyping

To identify whether PVN^hepatic^ demonstrated a typical neuropeptide phenotype, a subset of tissue was immunohistochemically labeled for three major neuropeptide phenotypes in PVN: oxytocin (OT), vasopressin (VP), and corticotropin release hormone (CRH) (11, 22). First, mice were deeply anesthetized by isoflurane until lack of withdrawal reflex (i.e., lack of tail pinch response), then brain tissue was collected after transcardiac perfusion and sectioned as detailed earlier. Serial free-floating sections containing PVN were rinsed with PBS. Nonspecific labeling was blocked with 10% donkey serum (Jackson ImmunoResearch, West Grove, PA) and a detergent wash was used to access the intracellular space (0.3% Triton in PBS) for 30 min. Sections were first incubated with primary antibody against either oxytocin (rabbit anti-oxytocin—AB911, MilliporeSigma, 1:500, 4°C) or vasopressin (rabbit anti-AVP—T-4565, Peninsula Laboratories, 1:1,000, 4°C) overnight followed by incubation with secondary antibody donkey anti-rabbit Alexa Fluor 568 (1:300; Thermo Fischer) for 4 h in a dark room at room temperature. Sections were then stained for PRV-152-mediated GFP using a primary antibody against GFP (chicken anti-GFP—ab13970, Abcam, 1:350, 4°C) followed by a secondary antibody donkey anti-chicken Alexa Fluor488 (1:300; Invitrogen, A-11039), in a dark room at room temperature. All sections were mounted on charged slides (Fisherbrand Superfrost Plus Microscope Slides, Fischer Scientific, NH) and coverslipped with Vectabond (with DAPI, VectorLaboratories, CA). To identify CRH neurons, CRH-Cre mice (23) were anesthetized with isoflurane (2%–5% in O_2_-inspired air) and stereotaxically injected bilaterally into PVN with AAV-hSyn-DIO-mCherry (250 nL/hemisphere; Addgene, 50459-AAVrg), following established coordinates (Antero-Posterior: 0.45 mm, Lateral: ±0.05 mm and Dorso-Ventral: −5.0 mm) (21). Allowing four weeks for viral inoculation, mice then received PRV-152 hepatic injections and brain tissue collected as detailed earlier. Serial free-floating sections were incubated with primary antibody rabbit anti-mCherry (Invitrogen, PA5-34974,1:1000, 4°C) overnight followed by incubation with secondary antibody donkey anti-rabbit Alexa Fluor 546 (1:300, Thermo Fischer) for 4 h in a dark room at room temperature, then stained for PRV-152-mediated GFP as detailed earlier.

### Imaging Acquisition and Analysis

All images were collected on either an Olympus BX43 captured with a Retiga R6 (Teledyne Imaging, Tucson, AZ) or All-in-One Fluorescence (Keyence BZ-X800E, Itasca, IL) microscope, using appropriate filters in ×4, ×10, and ×20. Images were adjusted on Fiji Software (ImageJ). Image acquisition parameters, including exposure time and illumination intensity, were identical across groups; brightness and contrast in all images were modified together and identically for figure presentation. For cell counting, images were analyzed on Fiji Software manually with the Cell Counter Notice plug-in.

### Hypothalamic Slice Preparation and On-Cell Patch-Clamp Recordings

To determine the impact of insulin signaling on PVN^hepatic^ neurons, animals were anesthetized with isoflurane to overdose and quickly decapitated under anesthesia 72 h after PRV-152 injection into the liver. Brains were rapidly removed and submerged in ice-cold, oxygenated (95% O_2_-5% CO_2_) artificial cerebrospinal fluid (aCSF), with composition (in mM): 124 NaCl, 3 KCl, 26 NaHCO_3_, 1.4 NaH_2_PO_4_, 11 glucose, 1.3 CaCl_2_, 1.3 MgCl_2_, and 1 kynurenic acid. All solutions had an osmolarity between 290 and 305 mOsmol/L and pH between 7.3–7.4. Brains were blocked to contain PVN, fixed on a vibratome stage (VT1000 S, Leica Biosystems, Buffalo Grove, IL), and cut in the coronal plane (300 µm). The slices were transferred to a holding chamber with aCSF that contains kynurenic acid, continuously oxygenated (95% O_2_-5% CO_2_) for 30 min at 32–34°C, followed by acclimation to room temperature. Slices were placed on an upright fixed-stage microscope with a differential interference contrast and infrared illumination. Slices were held in place with a nylon grid fixed to a platinum wire and were continuously perfused in the recording chamber with aCSF containing kynurenic acid (1 mM, Sigma-Aldrich, Saint Louis, MO) and picrotoxin (100 µM, GABA_A_ receptor antagonist). PVN^hepatic^ neurons were visualized under fluorescence illumination by a GFP filter. Firing frequency of neurons was examined in an on-cell patch-clamp recording configuration, only cells where at least one action potential could be detected were included for analysis to ensure cellular viability. No more than one cell was recorded per slice and no more than three cells per animal were included in a single group. Glass recording pipettes (5–7 MΩ, King Precision Glass, Claremont, CA) were pulled in a horizontal puller (P-97; Sutter Instrument Co., Novato, CO) and filled with a pipette solution containing (in mM): 130 K-gluconate, 1 NaCl, 5 EGTA, 10 HEPES, 1 MgCl_2_, 1 CaCl_2_ and 2–3 Mg-ATP, pH 7.3–7.4, adjusted with 5 M KOH.

### Drugs

All drugs were applied until a steady state was reached at which time analysis was done. Drugs included picrotoxin (100 µM), a GABA_A_ receptor antagonist, kynurenic acid (1 mM), a nonselective antagonist of *N*-methyl-d-aspartate (NMDA), α-amino-3-hydroxy-5-methyl-4-isoxazoleporpionic acid (AMPA) and kainate receptors, insulin (1 µM to 100 nU, Insulin Solution Human, Sigma-Aldrich, CAS-Number 11061-68-0, 19278), and the mammalian target of rapamycin (mTOR) inhibitor, rapamycin [RAPA; 200 nM, Sigma-Aldrich, CAS-Number 536123-88-9, first diluted in dimethyl sulfoxide (DMSO, Sigma-Aldrich D-4540), 0.01% of DMSO at final solution].

### Data Analysis

All electrophysiological data were analyzed offline using Clampfit 11.1(Molecular Devices). A minimum of 8–10 min following establishment of on-cell configuration was used to allow equilibration. Following equilibration, firing frequency was calculated for a 3-min duration. To determine the effect of drug bath application, firing frequency was again calculated for a 3-min duration 5 min after bath application of any drug.

### Statistical Analyses

All data were analyzed with Prism 9 (GraphPad Software, Boston, MA). Average data were expressed as means ± standard error of the mean (SE). Specific statistical analyses performed are noted in the text. Briefly, Student’s *t* tests were used when comparing two conditions. Paired and unpaired assessments were used when values were from the same subject or different subjects, respectively. To compare multiple conditions, when appropriate, a one-way (with or without repeated measures) ANOVA was used with post hoc Tukey’s multiple comparisons or two-way (with or without) repeated-measures ANOVA with post hoc Sidak’s multiple comparisons. To qualitatively determine changes in the distribution of firing frequency responses, a 10% change from baseline was considered an effect and neuronal responses were categorized as “increased,” “decreased,” or “no change” in activity after insulin application. *P* ≤ 0.05 was considered statistically significant.

## RESULTS

### DMV Retrograde Labeling after PRV Injection into Liver

**Figure 1.**
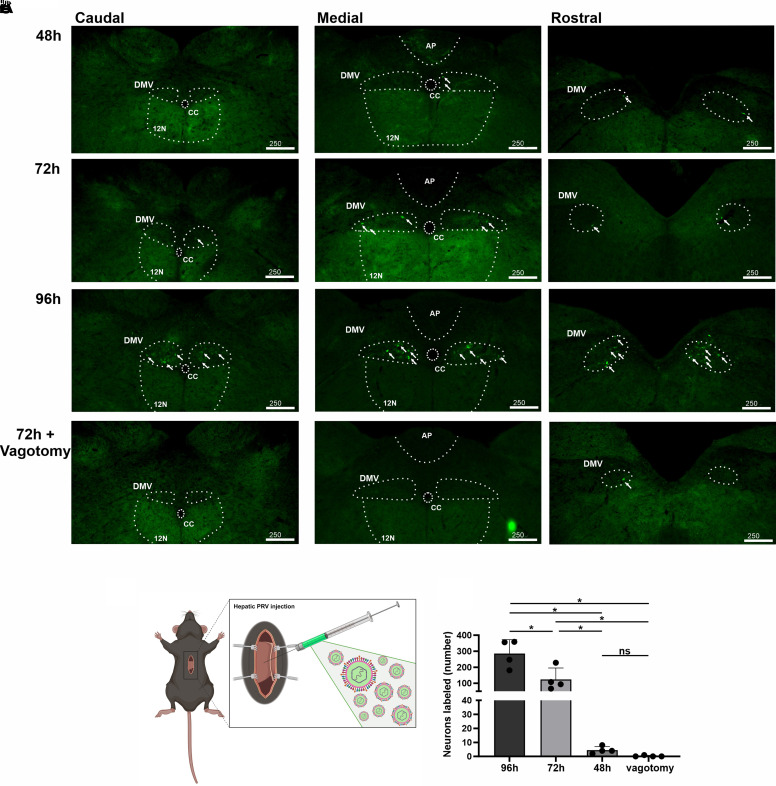
Dorsal motor nucleus of the vagus (DMV) retrograde labeling after pseudo-rabies virus (PRV) injection into liver. *A*: endogenous fluorescence of liver-related DMV neurons after retrograde labeling with PRV-Bartha 152 at caudal, medial, and rostral portions (vertical) after 48 h, 72 h, 96 h, and 72 h + vagotomy (horizontal). AP, area postrema; cc, central canal; 12N, hypoglossal nucleus. *B*: schematic drawing of surgery for liver injection of PRV-152. *C*: comparison of cell-counting of liver-related DMV neurons retrogradely labeled at 48 h, 72 h, 96 h after PRV-152 injection and after 72 h + vagotomy (*n* = 4 animals for all groups; two-way ANOVA with Tukey’s post-hoc). *P < 0.05.

To confirm the use of PRV to label parasympathetic motor neurons, the number of eGFP+ preganglionic parasympathetic motor neurons in DMV was examined 48 h, 72 h, and 96 h after injection of PRV-152 into the liver (Fig. 1, *A* and *B*). Since exhaustive labeling has been previously described (24), counts were restricted to DMV to qualitatively confirm labeling in DMV compared with PVN. One-way ANOVA revealed a significant effect of treatment [Treatment = Time postinjection or vagotomy; *F*(3,12) = 22.77; *P* < 0.0001] on labeled cell counts in DMV. Specifically, DMV contained a small number of eGFP+ neurons at 48 h (5 ± 1 neurons, *n* = 4; Fig. 1*C*), confirming that parasympathetic motor neurons in DMV are labeled by this technique early after hepatic injection. Later time points revealed a significant increase in the number of eGFP+ neurons in DMV at 72 h (124 ± 36 neurons, *n* = 4, *P* = 0.0467, Tukey’s post hoc test; Fig. 1*C*) and 96 h (285 ± 44 neurons, *n* = 4, *P* < 0.0001, Tukey’s post hoc test; Fig. 1*C*) postinjection compared with counts at 48 h. To confirm that eGFP+ labeling in this region required an intact left vagus, DMV eGFP+ neurons were also counted 72 h after hepatic injection in left cervical vagotomized animals. Vagotomy eliminated virtually all eGFP+ neurons in DMV 72 h after hepatic injection (0 ± 1 neuron, *n* = 4), which was significantly smaller than the number of eGFP+ neurons in DMV at 72 h (*P* = 0.0388; Tukey’s post hoc test) and 96 h (*P* < 0.0001; Tukey’s post hoc test) after hepatic injection in animals with an intact left vagus nerve.

### PVN Retrograde Labeling Occurs 24 H after DMV Labeling and Requires an Intact Vagus Nerve

**Figure 2.**
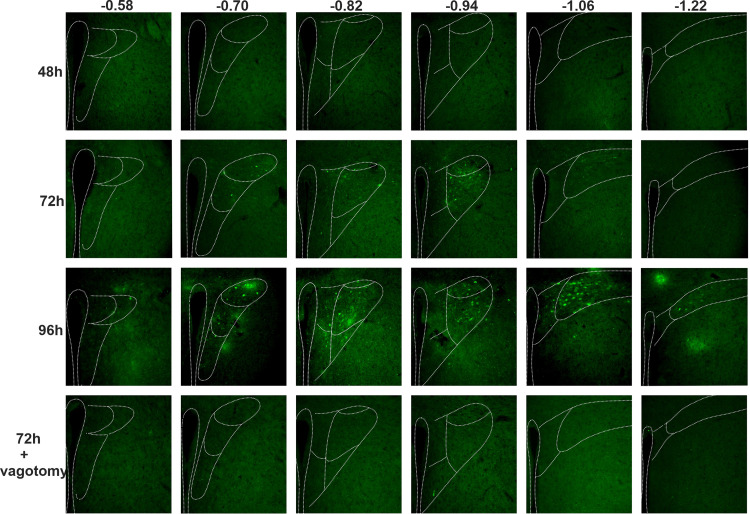
Paraventricular nucleus of the hypothalamus (PVN) retrograde labeling after pseudo-rabies virus (PRV) injection into liver. Endogenous fluorescence of hepatic-related PVN (PVN^hepatic^) neurons after retrograde labeling with PRV-152 across the rostrocaudal planes (vertical) after 48 h, 72 h, 96 h, and 72 h + vagotomy (horizontal).

**Figure 3.**
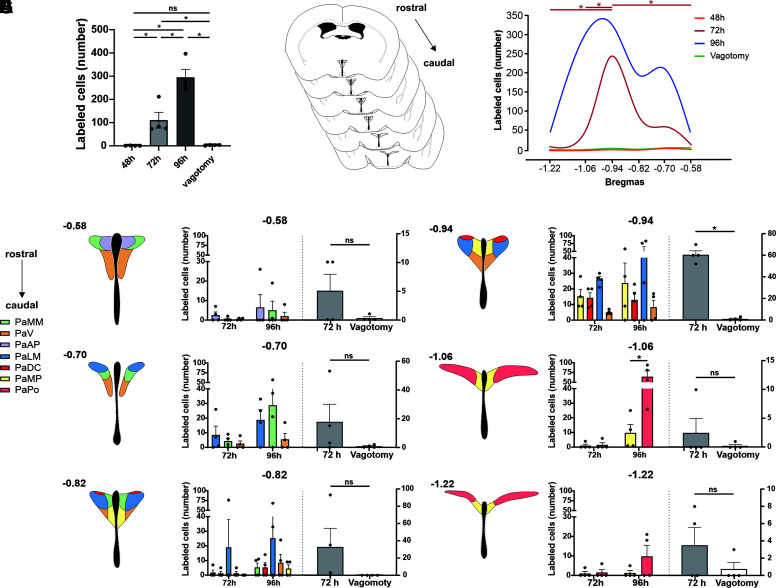
Quantification of paraventricular nucleus of the hypothalamus (PVN) retrograde labeling after pseudo-rabies virus (PRV) injection into liver. *A*: total number of PVN^hepatic^ neurons retrogradely labeled at 48 h, 72 h, 96 h after PRV-152 injection, and after 72 h + vagotomy (*n* = 4 animals for all groups; two-way ANOVA with Tukey’s pots hoc). *B*: schematic drawing of PVN bregma across all rostrocaudal planes. *C*: rostral to caudal distribution of neurons labeled at 48 h, 72 h, 96 h, and after 72 h + vagotomy. *D*–*I*: cell-counting of PVN^hepatic^ neurons labeled across all rostrocaudal planes, respectively (two-way repeated-measures ANOVA with Sidak’s post hoc), and by subpopulations and comparisons between 72 h versus vagotomy (unpaired two-tailed Student’s *t* test). PaMM, paraventricular hypothalamic nucleus anterior magnocellular part; PaV, paraventricular hypothalamic nucleus ventral part; PaAP, paraventricular hypothalamic nucleus anterior parvocellular part; PaLM, paraventricular hypothalamic nucleus lateral magnocellular part; PaDC, paraventricular hypothalamic nucleus dorsal cap; PaMP, paraventricular hypothalamic nucleus medial parvocellular part; PaPo, paraventricular hypothalamic nucleus posterior part. *P < 0.05.

To map a timeline for identification of parasympathetic-related neurons in PVN, eGFP+ neurons in PVN were identified and counted 48 h, 72 h, and 96 h after injection of PRV-152 into the liver (Fig. 1*B* and Fig. 2). One-way ANOVA revealed a significant effect of treatment [*F*(3,12) = 33.57; *P* < 0.0001] on labeled cell counts in the PVN. Congruent with direct PVN→DMV projections, PVN neurons did not express eGFP+ labeling 48 h after hepatic PRV-152 injection (1 ± 1 neurons, *n* = 4, Fig. 2 and Fig. 3*A*). Rather, PVN neurons first expressed eGFP 72 h after hepatic injection (111 ± 34 neurons, *n* = 4, Fig. 3*A*), which was significantly greater than expression at 48 h (*P* = 0.045; Tukey’s post hoc test) and occurred 24 h after eGFP+ expression in DMV. Expression of eGFP continued to increase at 96 h (294 ± 34 neurons, *n* = 4, Fig. 2) as the number of eGFP+ cells at 96 h was significantly greater than the number of eGFP+ cells at 72 h (*P* = 0.0009; Tukey’s post hoc test; Fig. 3*A*) and 48 h (*P* < 0.0001; Tukey’s post hoc test; Fig. 3*A*) posthepatic injection. To determine whether PVN contains putative parasympathetic neurons, PVN^hepatic^ labeling was also examined after a left cervical vagotomy (Fig. 2). Like DMV, vagotomy eliminated nearly all eGFP+ labeling in PVN 72 h after hepatic injection (3 ± 1 neurons, *n* = 4) compared with animals with intact vagi at 72 h (*P* = 0.0356; Tukey’s post hoc test; Fig. 3*B*) and 96 h (*P* < 0.0001, Tukey’s post hoc test; Fig. 3*A*) posthepatic injection.

### PVN^hepatic^ Labeling at 72 H Is Specific to Rostrocaudal Extension, but Not to Subregions

Since PVN is a heterogeneous population with multiple subregions, the distribution of PVN^hepatic^ neurons by rostrocaudal location and subregions were examined. At 72 h, PVN^hepatic^ labeling was present (regions with >2 cells) throughout PVN, with a significant effect of rostrocaudal location on number of labeled cells [*F*(5,18) = 4.859; *P* = 0.0055; one-way ANOVA, Fig. 3*C*]. Specifically, we observed significantly greater labeling in medial PVN at ∼−0.94 mm from bregma (61 ± 4 neurons, *n* = 4) than labeling at the rostrocaudal boundary of PVN, ∼−0.58 mm (4 ± 2 neurons, *n* = 4, *P* = 0.0129; Tukey’s post hoc test), −1.06 mm (3 ± 3 neurons, *n* = 4, *P* = 0.0112; Tukey’s post hoc test), and −1.22 mm (3 ± 3 neurons, *n* = 4, *P* = 0.0112; Tukey’s post hoc test) from bregma. By 96 h, PVN^hepatic^ labeling was robust throughout the entire rostrocaudal extent of PVN examined (Fig. 3, *C–I*), with no effect of rostrocaudal location on labeling. Since vagotomy abolished nearly all labeling in PVN 72 h after hepatic injection of PRV, rostrocaudal distribution of the number of PVN eGFP+ neurons from vagotomized and vagi-intact animals was also examined at this time point. Critically, vagotomy only significantly decreased labeling in medial PVN (−0.94 mm from bregma; vagi intact: 60 ± 4 neurons, *n* = 4, vs. vagotomized: 1 ± 1 neurons, *n* = 4; *P* < 0.0001, unpaired two-tailed Student’s *t* test; Fig. 3*G*). No other plane had statistically significant differences in eGFP+ cell counts between intact and vagotomized animals at 72 h post PRV-152 injection into hepatic tissue, potentially owing to the relatively low number of eGFP+ cells observed at these locations in vagi-intact animals at this time point.

To determine whether any subregions within PVN are more likely to contain parasympathetic neurons, all subregions of PVN were examined for eGFP+ neurons after hepatic injection with PRV-152 across the rostrocaudal extent of PVN (Fig. 3, *D–I*) (21). Two-way ANOVA revealed a significant interaction between PVN subregion and treatment only at −1.06 mm from bregma [Subregion × Treatment: *F*(3,24) = 11.29, *P* < 0.0001]. At this location (−1.06 mm from bregma), the posterior (PaPo) parvocellular subdivision (64 ± 15 neurons, *n* = 4) had significantly more labeled cells than medial (PaMP) parvocellular subdivision (10 ± 6 neurons, *n* = 4; <0.0001, Tukey’s post hoc test; Fig. 3*H*) of PVN at 96-h postinjection, potentially owing to the relatively larger area of PaPo compared with PaMP in this plane. At other rostrocaudal locations, there was no significant difference in number of eGFP+ neurons between subregions at 72-h or 96-h posthepatic injection.

### Phenotype Identification of PVN^hepatic^ Neurons

**Figure 4.**
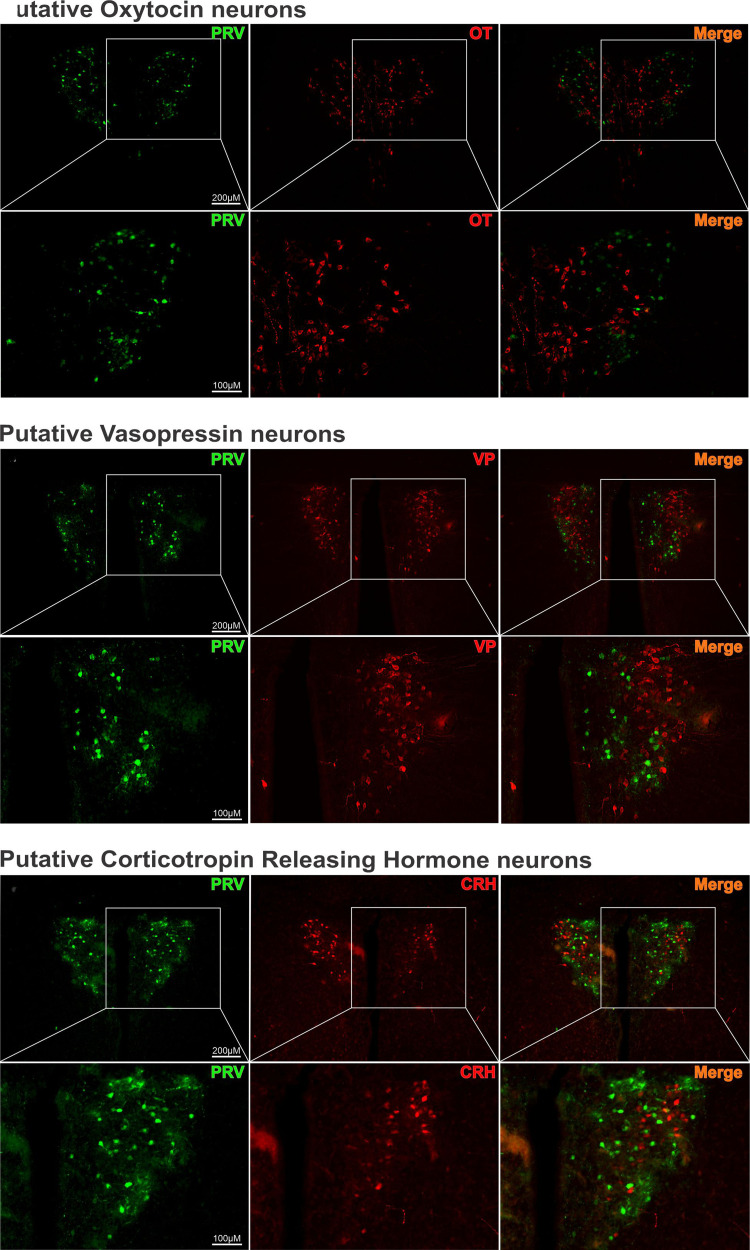
Phenotype identification of hepatic-related paraventricular nucleus of the hypothalamus (PVN^hepatic^) neurons. Neuropeptides immunostaining of PVN^hepatic^ neurons retrogradely labeled with PRV-152. Photomicrographs of coronal section of PVN^hepatic^ neurons (*A*, *G*, and *M*), oxytocin (OT; *B*), vasopressin (VP; *H*), and corticotropin-release hormone (CRH; *N*), and merged images (*C*, *I*, and *O*). *D*, *E*, *F*, *J*, *K*, *L*, *P*, *Q*, *R* represent higher magnification of the square depicted above it.

To identify the phenotype of PVN^hepatic^ neurons, we performed immunohistochemistry in a subset of tissues for three distinct neuropeptides: OT, VP, and CRH (Fig. 4). Despite qualitatively robust labeling of eGFP+ and OT+, no neurons colabeled at any bregma region examined (Fig. 4, *A–F*). This was also true of VP+ (Fig. 4, *G–L*) and CRH+ neurons (Fig. 4, *M*–*R*). Therefore, using this experimental approach, PVN^hepatic^ neurons are not likely to contain these three prominent neuropeptides.

### Insulin Increases PVN^hepatic^ Neurons Activity

**Figure 5.**
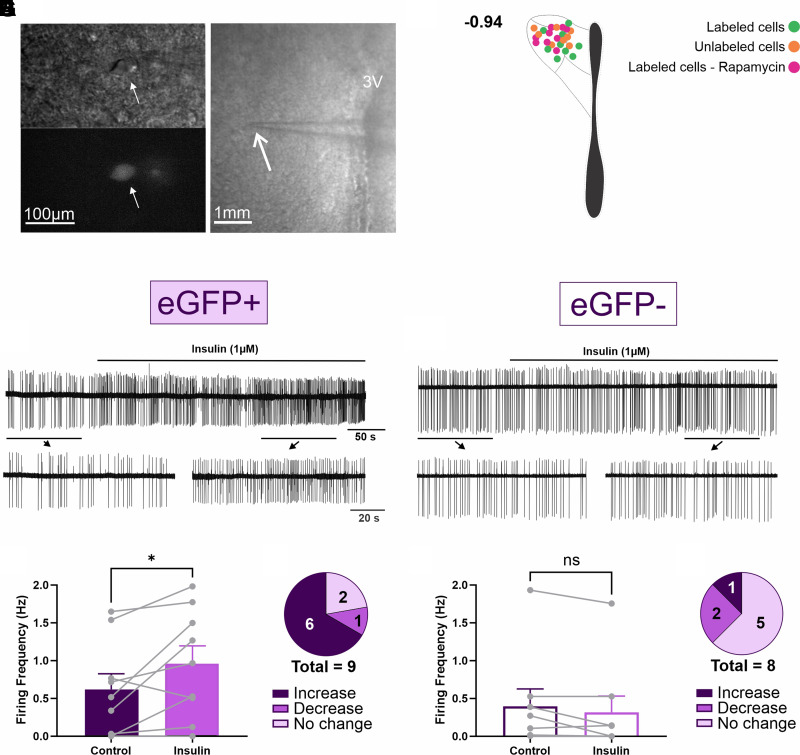
Insulin effects on hepatic-related paraventricular nucleus of the hypothalamus (PVN^hepatic^) neuron activity. *A*: representative image at high magnification of a single PVN^hepatic^ neuron under bright field and the same neuron under fluorescence. Additional image illustrates PVN and pipette at lower magnification. *B*: representative diagram depicting distribution of all neurons recorded. *C*: representative traces of a labeled neuron (enhanced green fluorescent protein, eGFP+) recorded before (control) and after insulin action. *D*: firing frequency comparison between periods control and insulin on PVN^hepatic^ neurons (*n* = 9 cells from 7 animals; paired two-tailed Student’s *t* test). *E*: Pie graph illustrating the distribution of individual responses to insulin (10% of change from control). *F*: representative traces of an unlabeled neuron (eGFP−) recorded before and after insulin action. *G*: firing frequency comparison between control and insulin on eGFP− PVN neurons (*n* = 9 cells from 7 animals; paired two-tailed Student’s *t* test). *H*: Pie graph illustrating distribution of individual responses to insulin (10% of change from control) in eGFP− neurons recorded. 3V, third ventricle; ns, not significant. *P < 0.05.

To determine the impact of insulin signaling on parasympathetic PVN^hepatic^ neurons, on-cell patch recordings were completed around bregma level −0.94, as vagotomy eliminated the vast majority of eGFP+ expression in PVN neurons in this region at 72-h posthepatic injection of PRV-152 (Fig. 5*C*). In PVN^hepatic^ neurons, bath application of insulin (1 µM) significantly increased firing frequency (0.961 ± 0.239 Hz, *n* = 9 cells) compared with baseline (0.618 ± 0.209 Hz, *n* = 9 cells from 7 animals; paired two-tailed Student’s *t* test, *P* = 0.0394, Fig. 5*D*). Furthermore, correlation of baseline firing frequency to insulin-mediated increase in firing frequency revealed an inverse relationship, whereby lower baseline firing frequency was associated with higher insulin-mediated increases in firing frequency (*Y* = −164.3 × *X* + 340.2; *P* = 0.0483; *R*^2^ = 0.5747; simple linear regression; data not shown). Qualitatively, the majority of PVN^hepatic^ neurons located at −0.94 mm from bregma responded to insulin application with an increase in firing frequency (67%; six of nine recorded cells; Fig. 5*E*). Only one cell responded with a decrease in firing frequency (11%), and two cells did not change firing frequency. To determine whether insulin responsivity is specific to PVN^hepatic^ neurons in this region, eGFP− neurons were also recorded before and after bath application of insulin (Fig. 5*F*). Interestingly, the firing frequency of unlabeled PVN neurons did not significantly change after insulin application (0.321 ± 0.214 Hz) compared with baseline (0.406 ± 0.229 Hz, *n* = 8 cells from 5 animals; paired two-tailed Student’s *t* test, *P* = 0.973, Fig. 5*G*). Unlike PVN^hepatic^ neurons, only one of eight (13%) unlabeled PVN neurons increased firing frequency. Most unlabeled neurons did not respond to insulin application (62%; 5 of 8 neurons); and two unlabeled PVN neurons decreased their firing frequency (25%) (Fig. 5*H*). Therefore, the ability of insulin to increase neuronal activity seems to be specific to PVN^hepatic^ neurons in this subregion using this approach.

### Insulin Effect on PVN^hepatic^ Neurons Is Dependent on mTOR Signaling

**Figure 6.**
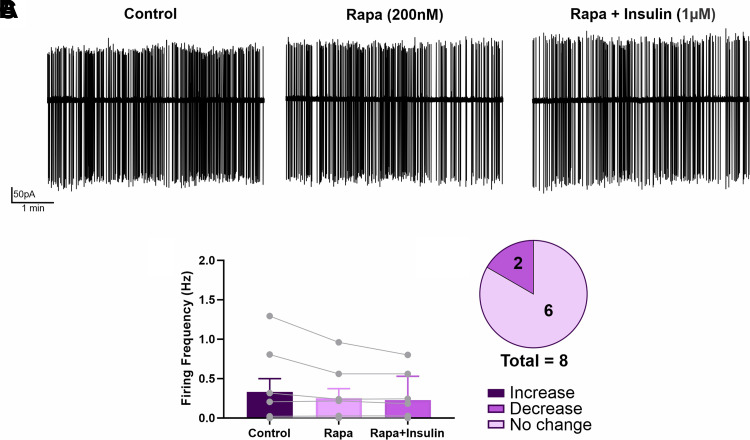
Insulin effect on hepatic-related paraventricular nucleus of the hypothalamus (PVN^hepatic^) neurons is dependent on mammalian target of rapamycin (mTOR) signaling. *A*: representative traces of a labeled neuron recorded during Rapamycin (RAPA) before bath insulin application (RAPA + Insulin). *B*: firing frequency comparison between control, RAPA, and RAPA + Insulin on PVN^hepatic^ neurons (*n* = 8 cells from 6 animals; one-way repeated-measures ANOVA with Tukey’s post hoc). *C*: illustration of the distribution of individual responses to insulin (10% of change from control) after pretreatment with RAPA.

Finally, to determine whether insulin-induced increases in PVN^hepatic^ neural activity require canonical intracellular insulin signaling pathways, a subset of PVN^hepatic^ neurons was pretreated with RAPA (200 nM) by bath application, followed then by insulin bath application in the continued presence of RAPA (Fig. 6*A*). Repeated-measures one-way ANOVA revealed no effect of drug treatment on neuronal firing frequency [*F*(1.065,7.458) = 2.649; *P* = 0.1447]. RAPA alone had no effect on firing frequency, with firing rate before (0.331 ± 0.169 Hz, range: 0.000–1.294 Hz; *n* = 8 cells) and after RAPA application (0.251 ± 0.123 Hz, range: 0.000–0.961; *n* = 8 cells; *P* = 0.2763, Tukey’s post hoc test; Fig. 6*B*) not statistically different. Interestingly, pretreatment with RAPA inhibited the ability of insulin to increase firing frequency in PVN^hepatic^ neurons, as firing frequency in RAPA + Insulin (0.227 ± 0.107 Hz) was not significantly different than baseline (*P* = 0.2938; Tukey’s post hoc test) and RAPA alone (*P* = 0.5035; Tukey’s post hoc test; Fig. 6*B*). Qualitatively, the majority of PVN^hepatic^ neurons (75%; 6 of 8 cells) showed “no change” in activity during insulin application, with no cells demonstrating an increase in activity, supporting the lack of insulin-mediated increase in firing frequency in the presence of RAPA. Similar to the minority of PVN neurons that were suppressed by insulin, two neurons demonstrated an insulin-induced decrease in firing frequency in the presence of RAPA (Fig. 6*C*). Taken together, mTOR intracellular signaling pathways must be active for insulin to increase firing frequency of PVN^hepatic^ neurons.

## DISCUSSION

The present study examined time-dependent retrograde labeling in PVN after PRV injection into the liver and the effect of insulin on parasympathetic circuit-related PVN neurons involved in central control of hepatic function. Hepatic-related DMV neurons were first labeled 48 h after liver inoculation with PRV, whereas PVN neurons were first labeled at 72 h. In addition, no labeling was found 72 h after hepatic injection when the left vagus nerve was transected. Together, these data implicate a neuronal population in PVN that can be labeled 72 h after hepatic injection of PRV as parasympathetic-related PVN^hepatic^ neurons. To determine whether insulin directly activates this population of PVN^hepatic^ neurons, on-cell patch-clamp techniques were used to investigate action potential firing in these neurons. Exogenous application of insulin increased firing frequency in putative PVN^hepatic^ parasympathetic-related neurons. This increase in neuronal activity required intracellular insulin signaling pathways dependent on mTOR, since rapamycin inhibited insulin-induced increases in PVN^hepatic^ neuronal firing. Importantly, insulin-induced increase in firing frequency was specific to PVN^hepatic^ neurons in the sampled population, as firing frequency was unchanged or decreased by insulin application in the majority (88%) of PVN neurons not expressing the eGFP. Taken together, these data contribute to the hypothesis that insulin activates pre-parasympathetic PVN^hepatic^ neurons via an mTOR-dependent pathway.

It is well established that parasympathetic premotor neurons, located in the brainstem, are involved in glycemic control (25). Moreover, physiological concentrations of insulin into hypothalamus are known to control nutrient availability by regulating energy balance and hepatic glucose production by using brainstem-dependent parasympathetic control of hepatic function (26). Although there is evidence that insulin can cross the blood-brain barrier (BBB) by saturable transport (4, 5, 27) and act in some brain areas, including hypothalamus to modulate autonomic nervous system activity, the location and mechanism of insulin’s actions on parasympathetic neurons upstream of brainstem motor output is not well established.

One potential site of insulin’s actions on parasympathetic motor drive is hepatic-projecting DMV neurons in the brainstem responsible for parasympathetic output to liver (28). Our results confirm previous reports that DMV neurons express PRV-152-eGFP when injected into liver, and this projection requires an intact left vagus nerve (19, 20, 24, 25), as a nontraditional, but established, characteristic of parasympathetic innervation to the liver since most other organs (i.e., heart, stomach, and intestines) are bilaterally innervated by both right and left vagi (29, 30).

Although insulin can provide minor inhibitory actions on DMV neurons (31), to our knowledge no study to date provides evidence of insulin modifying activity of DMV neurons with hepatic projections. However, insulin does have well-established actions in hypothalamus to regulate hepatic glucose production and this action requires parasympathetic motor innervation (27, 32–34). Therefore, the current study examined the potential for PVN, a critical hypothalamic region that integrates metabolic status with autonomic output as the site of insulin’s action for hepatic parasympathetic regulation (35). Although heterogeneous in nature, PVN projects monosynaptically to DMV (13, 14) and expresses insulin receptors (15). PVN also has a known role in the regulation of hepatic function (12, 16). To examine the existence of parasympathetic circuit-related neurons in PVN, initial experiments established a timeline for eGFP+ expression in PVN after PRV-152 injection into the liver parenchyma. PVN eGFP+ expression occurred 24 h after DMV neurons first express eGFP. This is consistent with PVN→DMV projections since an established characteristic of PRV is to infect an upstream neural population every 24 h (16, 36, 37). As with DMV, we further examined PVN eGFP+ expression after a left vagotomy and confirmed that this abolished nearly all eGFP+ labeling in medial PVN (−0.94 mm from bregma) at 72 h after hepatic injection of PRV-152. It remains possible that the current study under-reports the locations of bregma planes containing parasympathetic-related populations of neurons because of the relatively small sample size. Despite this small sample size, this location was the most prominently labeled and likely contains a significant number of neurons related to parasympathetic circuits. Taken together, PVN neurons labeled 72 h after hepatic infection with PRV-152 in this region (∼0.94 mm from bregma) were related to parasympathetic motor output, likely through direct connections to DMV. Further characterization of the spatial distribution of PVN^hepatic^ neurons revealed subregion (e.g., PaMM, PaV, PaAP, PaLM, PaDC, PaMP, and PaPo) distribution at specific rostrocaudal locations (21). In rostrocaudal locations containing labeled cells, retrogradely labeled neurons were located in all subdivisions and the number of eGFP+ (putative PVN^hepatic^) neurons was for the most part similar between PVN subdivisions.

We further sought to determine whether putative PVN^hepatic^ neurons expressed any major peptide phenotypes common in PVN (38). PVN is composed of two main classes of neurons, magnocellular and parvocellular (11). Although magnocellular neurons project primarily to the posterior pituitary gland, parvocellular neurons have a diversity of projections, including projections to sympathetic regulating regions (i.e., rostral ventral lateral medulla and intermediolateral cell column) and parasympathetic regulating regions (i.e., DMV and nucleus ambiguus). Parvocellular PVN neurons are also phenotypically diverse, expressing immunoreactivity for VP, OT, and CRH among others (22). Again, using PRV-152 retrograde tracing paired now with immunohistochemical approaches, eGFP+ neurons in PVN were examined for three main neuropeptides (VP, OT, and CRH). Despite robust eGFP+ and neuropeptide positive labeling throughout PVN, putative PVN^hepatic^ neurons did not colocalize with VP+, OT+, or CRH+ neurons. Nonetheless, PVN^hepatic^ neurons may express other neuropeptides not examined here. It is also possible that PVN^hepatic^ neurons are glutamatergic or GABAergic using primarily fast neurotransmission. Since most GABAergic neurons related to PVN activity do not reside in the PVN areas examined here, but rather they reside in the area surrounding PVN (39, 40), it is unlikely that PVN^hepatic^ neurons are GABAergic in nature. GABAergic varicosities in PVN do not express insulin receptors (41), providing further evidence that PVN^hepatic^ neurons are not likely GABAergic. Taken together, we speculate that PVN^hepatic^ neurons are glutamatergic, a common phenotype in PVN (17, 42). Glutamatergic projections from PVN to DMV have previously been described (18) and are suggested as the primary neurotransmitter for PVN communication to DMV (17). Although this work builds a foundation for understanding PVN^hepatic^ circuitry as it relates to parasympathetic output to liver, more work must be done to evaluate the neurotransmission properties and potential neuroplasticity of communication between PVN^hepatic^ neurons and DMV motor output.

To determine whether PVN^hepatic^ neurons serve as a site for parasympathetic-mediated insulin actions on hepatic function, PVN^hepatic^ neuronal responses to exogenous insulin application were examined. Insulin induced a robust increase in firing frequency in PVN^hepatic^ neurons, even when traditional fast synaptic neurotransmission was blocked. This is consistent with PVN^hepatic^ as a central site where insulin directly impacts neuronal activity, independent of upstream synaptic neurotransmission. Importantly in the present study, insulin-mediated increases in firing frequency were specific to labeled PVN^hepatic^ neurons as a subgroup of GFP− neurons (e.g., those that did not demonstrate endogenous fluorescence) did not demonstrate increases in firing frequency during insulin application. Previous work identified that insulin hyperpolarizes PVN neurons through ATP-sensitive potassium channels (43). In our experiments, we observed an insulin-mediated decrease in firing frequency, as would be expected during membrane hyperpolarization, in ∼11% of PVN^hepatic^ neurons (and this percentage was not altered by RAPA). However, this was not the primary response of this projection class. Moreover, a recent study confirmed that PVN^hepatic^ neurons related to sympathetic regulating central circuits were unaffected by insulin application ex vivo (44). As such, the two labeled cells that did not respond to insulin could have been sympathetic-related PVN^hepatic^ neurons. Although an additional synapse in the sympathetic arm likely precludes robust sympathetic-related PVN labeling at 72 h, vagotomized animals did have a small number of neurons labeled in PVN at 72 h that could represent a small population of sympathetic related PVN^hepatic^ neurons, likely synapsing directly in the intermediolateral cell column.

Insulin can induce neuronal excitation (45, 46), including in hypothalamic neurons (47). Since the present study eliminated traditional, fast neurotransmission (i.e., glutamate and GABA), it is likely that this increase in firing frequency results from a change in intrinsic membrane properties. Accordingly, insulin can also increase the firing rate of olfactory sensory neurons (48), similar to what we observed in our experiments. This increase has been postulated to occur through phosphorylation-mediated inhibition of Kv1.3 channels (49), which can promote increased membrane excitability. As PVN contains Kv1.3 (48), it is possible that the insulin-mediated increase in firing frequency we observed in PVN^hepatic^ neurons is due to insulin-dependent inhibition of Kv1.3. However, the current study cannot rule out insulin impacting presynaptic terminal release of other neuromodulators such as α-melanocyte stimulating hormone or neuropeptide Y. Regardless of mechanism(s) of action (i.e., intrinsic membrane property or presynaptic neuromodulator release), our results indicate a novel pathway responsible for the actions of insulin in central circuits and demonstrate a new mechanism of action specifically for parasympathetic-mediated hepatic function.

To further elucidate intracellular pathways responsible for the actions of insulin on PVN^hepatic^ neurons, we examined the canonical insulin intracellular signaling pathway using the mammalian target of rapamycin (mTOR) (50). Bath application of RAPA, an inhibitor of mTOR1 and mTOR2 activity, prevented insulin-induced increase in firing frequency of PVN^hepatic^ neurons. Surprisingly, the action of insulin (and subsequent inhibition of its activity with RAPA) is rapid in nature (∼5 min) considering mTOR’s activity as a gene regulator. However, activation of mTOR through insulin administration (51) or amino acid supplementation (52) can occur within minutes. Although outside the scope of the current manuscript, mTOR can alter several types of receptors through cascades of phosphorylation that occur when insulin receptors are activated (53). Considering the rapid activation of mTOR following insulin administration, it is possible that insulin-mediated mTOR activation could drive phosphorylation-mediated inhibition of voltage-gated potassium channels that may ultimately underlie the insulin-mediated increases in firing frequency that is blocked by RAPA.

In addition, mTOR activity has a number of reciprocal inhibitory interactions with other intracellular signaling pathways. Therefore, it is possible that when RAPA inhibits mTOR activity, it activates alternative intracellular signaling cascades. For example, adenosine monophosphate-activated protein kinase (AMPK) activity can be restrained by mTOR activation, and this can occur in hypothalamic neurons (54). AMPK activation (as would occur if mTOR was inhibited) not only can decrease overall firing frequency but can cause a hyperpolarizing shift for voltage-gated potassium channels to reduce the impact of other excitatory drives (55), such as might occur when insulin receptors are activated. Therefore, it is possible that parasympathetic-related PVN^hepatic^ neurons maintain levels of mTOR expression to offset the activation of AMPK activity to allow for increased cellular activity during insulin receptor activation. Although it remains possible that RAPA simply inhibited the ability of the cell to respond, RAPA itself did not alter PVN^hepatic^ neuron activity (Fig. 6, *A* and *B*) and neurons with the lowest firing frequency responded more robustly to insulin application (Fig. 5*D*) suggesting that low activity itself does not preclude increases in firing frequency during insulin application. Therefore, it is likely that this effect was specific to insulin receptor activation. Taken together, these results provide evidence that insulin acts on PVN^hepatic^ neurons to activate a PVN→DMV→liver circuit through an mTOR-dependent intracellular insulin receptor signaling pathway. Future directions should examine in more detail the impact of mTOR on central insulin signaling, including possible interactions with other intracellular signaling cascades.

### Conclusions

In conclusion, despite insulin’s well-established peripheral mechanisms of action to regulate glucose homeostasis through actions on the liver, the present study combined with previous work, confirms that insulin also acts on central neural networks to impact hepatic function. We provide evidence that insulin activates putative parasympathetic-related PVN^hepatic^ neurons through mTOR-dependent signaling pathways. Since these neurons are likely glutamatergic in nature, insulin-dependent increases in action potential firing would release glutamate into DMV increasing the likelihood of DMV excitation. Indeed, increased DMV activity and ultimately parasympathetic motor output decreases hepatic glucose production, glycogenolysis and hepatic glucose release (26, 34). Taken together, we mechanistically identified a novel hypothalamic to brainstem pathway capable of mediating insulin-dependent activation of parasympathetic motor output.

### Critical Considerations

Finally, it is crucial to note that although the PVN is situated around the third ventricle, it does not qualify as a circumventricular organ; rather, it is densely vascularized (56). As discussed previously, insulin can cross the BBB, and it is possible that it can reach PVN neurons by crossing blood vessels in the region. Insulin levels in the cerebrospinal fluid (CSF) are lower than in the blood (57), however, its level in CSF increases after insulin is administered intravenously (58, 59). The concentration of insulin used in this study was 1 µM, which is equivalent to 100 nU as previously used (31, 59) to mimic postprandial insulin levels.

## DATA AVAILABILITY

Data will be made available upon reasonable request.

## GRANTS

This work was supported by NIH R01 HL157366 (to C.R.B.), FAPESP No. 2021/10017-4 (to K.M.d.S.) and No. 2023/08762-9 (to V.R.A.). V.R.A. is a Research Fellow of The National Council for Scientific and Technological Development (CNPq) under Grant No. 305570/2023-4.

## DISCLOSURES

Authors have not used any generative artificial intelligence tools in the preparation of this manuscript. None of the other authors has any conflicts of interest, financial or otherwise, to disclose.

## AUTHOR CONTRIBUTIONS

K.M.d.S., V.R.A., and C.R.B. conceived and designed research; K.M.d.S. performed experiments; K.M.d.S. and S.E.S. analyzed data; K.M.d.S., S.E.S., V.R.A., and C.R.B. interpreted results of experiments; K.M.d.S., S.E.S., V.R.A., and C.R.B. prepared figures; K.M.d.S., V.R.A., and C.R.B. drafted manuscript; K.M.d.S., S.E.S., V.R.A., and C.R.B. edited and revised manuscript; K.M.d.S., S.E.S., V.R.A., and C.R.B. approved final version of manuscript.
